# Defensive Properties of Ginsenoside Re against UV-B-Induced Oxidative Stress through Up-Regulating Glutathione and Superoxide Dismutase in HaCaT Keratinocytes

**Published:** 2018

**Authors:** Daehyun Shin, Hee Won Moon, Yuri Oh, Kyunghoon Kim, Dae-Duk Kim, Chang-Jin Lim

**Affiliations:** a *Research and Development Center, Cosmocos Corporation, Incheon 21698, Korea. *; b *Department of Biochemistry, College of Natural Sciences, Kangwon National University, Chuncheon 24341, Korea. *; c *Department of Biological Sciences, College of Natural Sciences, Kangwon National University, Chuncheon 24341, Korea. *; d *College of Pharmacy and Research Institute of Pharmaceutical Sciences, Seoul National University, Seoul 08826, Korea.*

**Keywords:** Ginsenoside Re, Reactive oxygen species, Matrix metalloproteinase, Glutathione, Superoxide dismutase, UV-B-irradiated HaCaT

## Abstract

Ginseng is now used worldwide as a traditional Oriental medicine. Ginsenosides, also known as ginseng saponins, are responsible for most pharmacological efficacies of ginseng. This work aimed to assess the novel skin anti-photoaging potential of ginsenoside Re (Re), a protopanaxatriol-type ginsenoside, by analyzing reactive oxygen species (ROS), pro-matrix metalloproteinase-2 (proMMP-2) and -9 (proMMP-9), total glutathione (GSH), superoxide dismutase (SOD), and cellular viability in UV-B-irradiated HaCaT keratinocytes. When HaCaT cells were pretreated with Re prior to UV-B irradiation, Re significantly suppressed the UV-B-induced ROS elevation. It was also able to attenuate the UV-B-induced proMMP-2 and -9 elevations at both activity and protein levels. Re was capable of overcoming the UV-B-reduced total GSH content and SOD activity in concentration-dependent ways. Under the experimental conditions used, Re could interfere with cellular viabilities in neither non-irradiated nor UV-B-irradiated keratinocytes.

## Introduction

**Figure 1:**
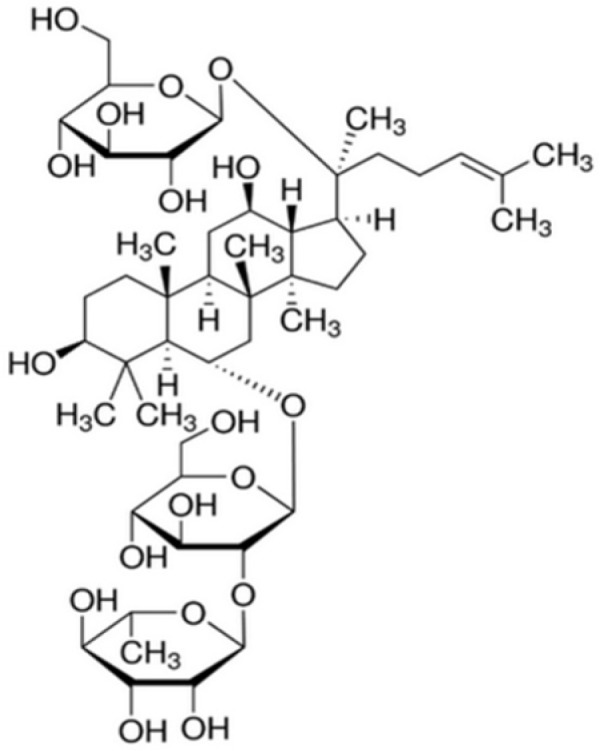
The chemical structure of ginsenoside Re (Re).

Ginsenosides are well-known triterpene glycosides which have been identified to be active ingredients for diverse pharmacological actions of ginseng. Ginseng, referring as to the dried roots of *Panax ginseng* C.A. Meyer (Araliaceae) used in the Oriental traditional medicine for a long time, has been gaining worldwide popularity over recent years. Its pharmacological actions gradually attract more and more interest. Ginsenosides, also known as ginseng saponins, are categorized into the three major classes, such as protopanaxadiol (PPD)-, protopanaxatriol (PPT)- and oleanolic acid-type ginsenosides, according to the chemical structures of their aglycones called sapogenins. Ginsenoside Re (Re, Figure 1) is a PPT-type ginsenoside which is one of the main ginsenosides abundantly present in ginseng.

Re has been already shown to have antioxidant and antioxidant-related properties in diverse cell types. When chick embryonic cardiomyocytes are exposed to hydrogen peroxide and antimycin A, a mitochondrial electron transport chain site III inhibitor which enhances endogenous oxidative stress, Re attenuates intracellular reactive oxygen species (ROS) levels and diminishes cell death, implying its potent antioxidant capacity which protects cardiomyocytes from oxidant injury by both exogenous and endogenous oxidants (1). Re plays protective roles against the occurrence of oxidative stress due to a depletion of glutathione (GSH) in streptozotocin-induced rats, lowers blood glucose and lipid levels, and restores the levels of both GSH and malondialdehyde in the eye and kidney, suggesting its effectiveness as an antidiabetic agent (2). Re protects against compound 48/80-induced acute gastric mucosal lesions in rats, through its stimulatory action on gastric mucus synthesis and secretion and its inhibitory actions on neutrophil infiltration and enhanced lipid peroxidation in the gastric mucosal tissue (3). Re exerts a plausible beneficial effect on neuroinflammatory events in neurodegenerative diseases via protecting against lipopolysaccharide-treated microglial cells through phosphor-p38, inducible nitric oxide synthase, and cyclooxygenase 2 signaling pathways (4).

Oxidative stress is defined as a redox imbalance between an excess of production of free radicals and a defect in antioxidant defense. Hydrogen peroxide, malondialdehyde, GSH, superoxide dismutase (SOD), and GSH peroxidase are its prominent markers. UV-B-induced oxidative stress induces the production of ROS, resulting in lipid peroxidation and additional types of DNA damage (5). Skin cells, including keratinocytes and fibroblasts, defend against oxidative stress through the cooperation of chemical and enzymatic antioxidants. Exposure of the skin to UV-B radiation and to oxidant stimuli induces a rapid production of intracellular ROS, detected as attenuation of catalase activity and vitamin E and reduced GSH levels, which in turn triggers keratinocyte growth factor receptor activation and internalization, similar to those induced by keratinocyte growth factor (6). Since chronic and repeated exposure of the skin to UV-B radiation may lead to photoaging and skin cancer, the defense against UV-B-induced oxidative stress gradually becomes even more important, particularly in older people.

Until recently, the beneficial properties of Re on the human skin have not been clearly recognized in pharmacological aspects. We could preliminarily assess the defensive effects of Re against UV-B-induced oxidative stress in HaCaT keratinocytes. In this report, we demonstrate that Re is able to suppress the UV-B-induced ROS and proMMP-2 and -9 elevations and enhances the UV-B-reduced total GSH contents and SOD activities in HaCaT keratinocytes, which implies its potential skin anti-photoaging properties.

## Experimental

*Materials and reagents*

Ginsenoside Re (Re, purity ≥ 98%) was bought from Ambo Institute (Seoul, Korea). Bradford reagent, 3-(4,5-dimethylthiazol-2-yl)-2,5-diphenyltetrazolium bromide (MTT), 2’,7’-dichlorodihydrofluorescein diacetate (DCFH-DA), dihydroethidium (DHE), 5,5›-dithiobis(2-nitrobenzoic acid) **(**DTNB), glutathione reductase (GR), reduced glutathione (GSH), NADPH, cytochrome c, xanthine, and xanthine oxidase were purchased from Sigma-Aldrich Chemical Co. (St Louis, MO, USA). Fetal bovine serum (FBS), Dulbecco’s modified Eagle’s medium (DMEM) and penicillin-streptomycin were obtained from HyClone Laboratories Inc. (Logan, UT, USA). Cell lysis buffer was from Promega Korea (Seoul, Korea). All other chemicals used were of the highest grade commercially available.

*Cell line and culture*

Immortalized HaCaT keratinocytes (ATCC, Manassas, VA, USA) were cultured in DMEM with 10% heat-inactivated FBS, 100 units/mL penicillin and 100 μg/mL penicillin-streptomycin in a humidified atmosphere with 5% CO_2_ at 37 °C. Prior to the treatments, 1 x 10^5^ HaCaT keratinocytes were seeded on 24-well plates and cultured overnight, washed twice with 1 mL phosphate-buffered saline (PBS), and replaced with 1 mL FBS-free medium. After UV-B irradiation without or with Re treatment, HaCaT cells were grown under the same growth conditions.

*UV-B irradiation*

An ultraviolet lamp (peak emission, 312 nm; model VL-6M, Vilber Lourmat, Marine, France) was used as a UV-B source. The UV-B dose was monitored with a radiometer (model VLX-3W, Vilber Lourmat, Marine, France) equipped with a sensor (bandwidth, 280 to 320 nm; model CX-312, Vilber Lourmat, Marine, France). The cultured HaCaT keratinocytes were irradiated with 70 mJ/cm^2^ UV-B radiation. The applied UV-B dose, which was determined to be sufficient to induce oxidative stress, took a 2.5 min exposure under the experimental conditions used.

*Cellular lysate preparation*

After adherent cells were washed twice with PBS and stored on ice for 5 min, they were physically detached using a cell scraper and obtained by centrifugation at 15,000 rpm for 10 min. The cell pellets were resuspended in cell lysis buffer [25 mM Tris-phosphate (pH 7.8), 2 mM 1,2-diaminocyclohexane-N,N,Nv,Nv-tetraactic acid, 2 mM dithiothreitol, 10% glycerol, 1% Triton X-100] and stored for 30 min on ice. Cellular lysate was taken after centrifugation at 15,000 rpm for 15 min. Proteins in cellular lysates were quantitated according to Bradford protein assay (7) using bovine serum albumin as a standard.

*Quantitation of intracellular ROS*

HaCaT keratinocytes were pretreated with varying concentrations (0, 5, 12 or 30 μM), chosen from a preliminary test, of Re prior to the irradiation with 70 mJ/cm^2^ UV-B radiation. Two fluorescent ROS probes, DCFH-DA and DHE, were used to quantitate the intracellular ROS levels. To fluorometrically determine the ROS levels in HaCaT cells, a redox-sensitive fluorescent probe DCFH-DA, which produces the fluorescent 2’,7’-dichlorofluorescein (DCF; λ_excitation_ = 485 nm, λ_emission_ = 530 nm) upon enzymatic reduction and subsequent oxidation by ROS, was used as previously described (8). After the treatment with Re and/or 20 µM DCFH-DA for 30 min at 37 ^o^C, the cells were washed twice with 1 mL FBS-free medium. 

The cells were resuspended in 1 mL FBS-free medium and irradiated with 70 mJ/cm^2^ UV-B radiation. The ROS levels were promptly determined by Multi-Mode Microplate Reader (Synergy^TM^ Mx, BioTek Instruments, Winooki, VT, USA). DHE, other fluorescent ROS probe, was utilized in a similar manner.

*Cell viability assay*

The cell viabilities of HaCaT keratinocytes in the presence of Re were determined by MTT assay used to assess metabolic activity (9). Cells were subjected to Re for 30 min. The cells, after removing the medium, were treated with 5 µg/mL MTT in medium for 4 h. The cells were then lysed with dimethyl sulfoxide, and the amount of formazan, produced from the reduction of MTT by the mitochondria of living cells, was quantitated by the absorbance at 540 nm.

*Gelatin zymography assay*

The proMMP-2 and -9 gelatinolytic activities in conditioned media was detected using zymographic analysis (10). Cells were incubated for 24 h at 37 °C, and washed twice with 1 mL PBS. The cells in 1 mL FBS-free medium were pretreated with Re for 30 min and irradiated with 70 mJ/cm^2^ UV-B radiation. The conditioned medium, obtained from the irradiated culture incubated for 24 h at 37 °C, was separated on 10% (w/v) SDS-PAGE gel impregnated with 1 mg/mL gelatin under non-reducing condition. The proteins in the gel were renatured by shaking with 2.5% Triton X-100 at room temperature for 30 min, which was repeated two times, and incubated in incubation buffer (50 mM Tris buffer, pH 7.8, 5 mM CaCl_2_, 0.15 M NaCl, 1% Triton X-100) for 24 h. After the gel was stained with 0.1% Coomassie Brilliant Blue R-250, the gelatin-degrading enzyme activities were convinced as clear zones against a blue background. proMMP-2 and -9 activity bands were determined in accordance with their molecular masses (72 kDa, 92 kDa), which were estimated by molecular mass markers.

*Western blot analysis*

Western blot analysis was performed to detect proMMP-2 and -9 in cellular lysate using anti-MMP-2 (ALX-210-753, Enzo Life Sciences, Farmingdale, NY, USA) and anti-MMP-9 (3852S, Cell Signaling Technology, Danvers, MA, USA) antibodies as primary antibodies. GAPDH, as an internal standard, in cellular lysate was detected using anti-GAPDH antibody (LF-PA0212, AbFrontier, Seoul, Korea). Cellular lysate was separated on 10% (w/v) SDS-PAGE and electrotransferred to PVDF transfer membrane. 

The membrane was blocked with blocking buffer (2% BSA in 1x TBS-Tween 20), probed with primary antibody overnight at 4 °C, incubated with secondary antibody (goat anti-rabbit IgG-pAb-HRP-conjugate; ADI-SAB-300, Enzo Life Sciences, Farmingdale, NY, USA) for 1 h at room temperature, and developed using an enhanced West-save up ^TM^ (AbFrontier, Seoul, Korea).

*Determination of total GSH*

Total GSH contents in cellular lysates were determined using an enzymatic recycling assay based on GR (11). The reaction mixture (200 μL), which contained 175 mM KH_2_PO_4_, 6.3 mM EDTA, 0.21 mM NADPH, 0.6 mM DTNB, 0.5 units/mL GR, and cellular lysate, was incubated at 25 °C. Absorbance at 412 nm was monitored using a microplate reader. Total GSH was expressed as μg/mg protein.

*Determination of SOD activity*

Total SOD activities in cellular lysates were spectrophotometrically determined as the reduction of cytochrome c with xanthine/xanthine oxidase system (12). The reaction mixture (200 μL) contained 50 mM phosphate buffer (pH 7.4), 0.01 units/mL xanthine oxidase, 0.1 mM EDTA, 1 μM catalase, 0.05 mM xanthine, 20 μM cytochrome c and cellular lysate. A change in absorbance at 550 nm was monitored.

*Statistical analysis*

The results were expressed as mean ± SD. Differences between experimental groups were analyzed using one-way ANOVA followed by post-hoc Tukey HSD test in JMP statistical software for multiple comparisons. A *P*-value less than 0.05 was considered statistically significant.

## Results

*Suppression on UV-B-induced ROS elevation*

**Figure 2:**
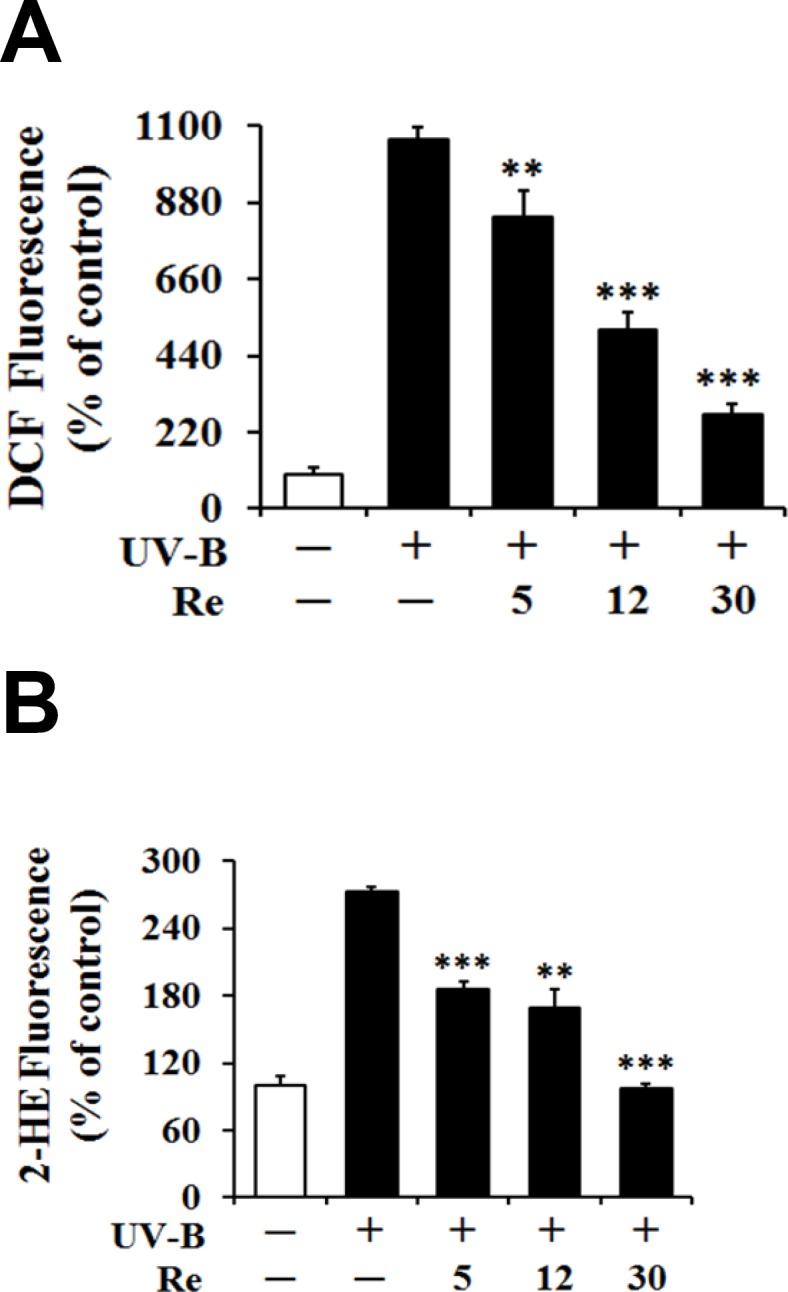
Suppressive effects of Re on the reactive oxygen species (ROS) levels in HaCaT keratinocytes under the irradiation with 70 mJ/c$m^{2}$UV-B. The HaCaT cells were incubated for 24 h, and pretreated with the indicated concentrations (0, 5, 12 and 30 μM) of Re for 30 min before irradiation. The ROS level was determined by DCFH-DA (A) and DHE (B) using fluorometry. The ROS level was represented as DCF and 2-HE fluorescences, arbitrary units expressed as % of control. **P < 0.01; ***P < 0.001 versus the non-treated control (UV-B irradiation alone

As shown in Figures 2A and 2B, the UV-B irradiation alone, in the absence of Re pretreatment, could bring about significant elevation in the ROS level over that in the non-irradiated control cells. Re could concentration-dependently attenuate the UV-B-induced ROS elevation (Figure 2A). 

Re at the concentrations of 5, 12 and 30 μM reduced the UV-B-induced ROS enhancement to 78.2%, 46.6% and 25.6% of that from the UV-B irradiation alone, respectively (Figure 2A). When the intracellular ROS levels were determined using DHE, Re also showed the attenuating effects on the UV-B-induced ROS elevation (Figure 2B). Re at 5, 12 and 30 μM could diminish the UV-B-induced ROS elevation to 68.3%, 62.1% and 35.6% of that from the UV-B irradiation alone, respectively (Figure 2B). Taken together, Re plays a suppressive role on the UV-B-induced ROS elevation in HaCaT keratinocytes, possibly suggesting its antioxidative activity.

*Nontoxicity on cellular viability*

**Figure 3:**
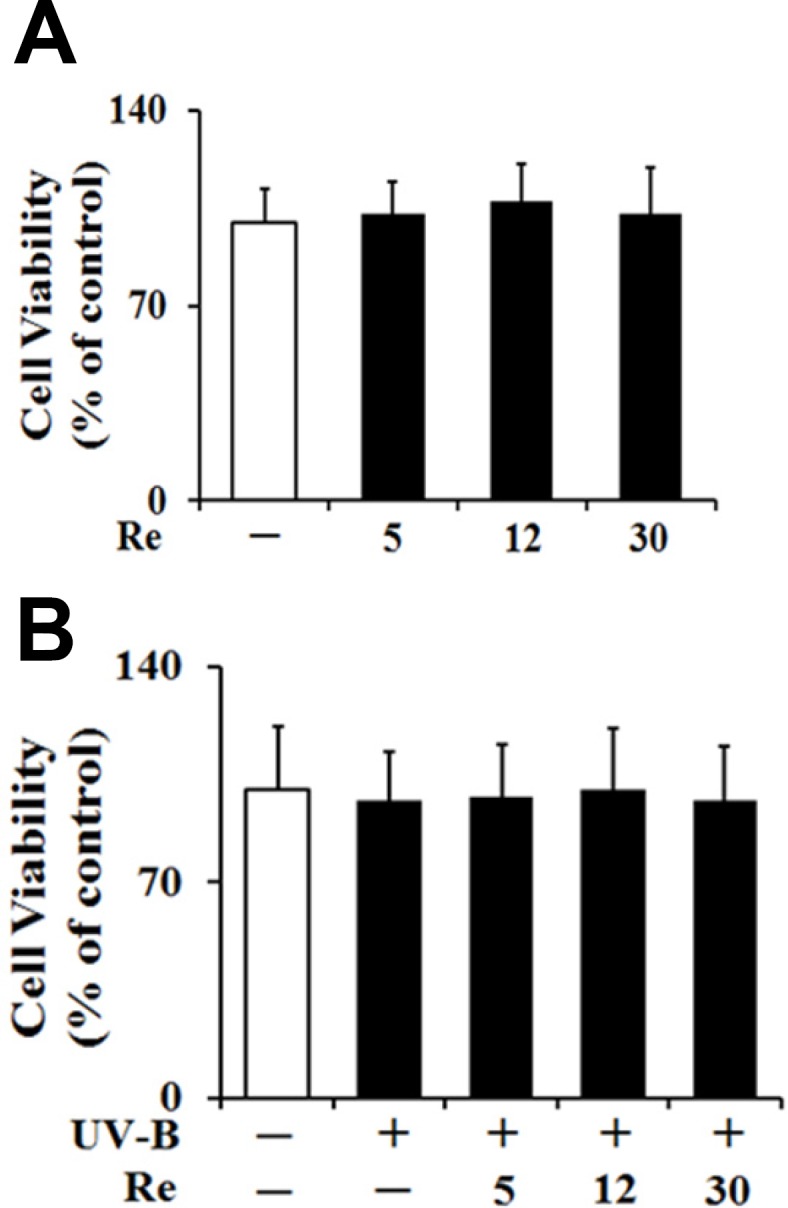
Effects of Re on the cellular viabilities in HaCaT cells in the absence (A) or presence (B) of the irradiation with 70 mJ/cm^2^ UV-B. The HaCaT cells were incubated for 24 h, and pretreated with the indicated concentrations (0, 5, 12 and 30 μM) of Re for 30 min before irradiation, if necessary. The viable cell numbers, expressed as % of control, were determined using MTT assay. Each bar shows the mean ± SD of the three independent experiments repeated in triplicate

To test whether Re and UV-B radiation at the experimental conditions exhibit cytotoxicities on HaCaT keratinocytes or not, their effects on the cellular viabilities of HaCaT keratinocytes were determined using MTT assay. Re, in the absence of UV-B irradiation, displayed no cytotoxicities and gave rise to the similar cellular viabilities, compared with the non-treated control (Figure 3A). As shown in Figure 3B, the UV-B irradiation at 70 mJ/cm^2^, in the absence of Re pretreatment, couldn’t cause a modulation in the cellular viabilities of HaCaT keratinocytes, and they were maintained to be similar to those of the non-irradiated cells. Re was not able to alter the cellular viabilities of HaCaT keratinocytes under UV-B irradiation (Figure 3B). 

Collectively, Re exhibit no cytotoxicity on HaCaT keratinocytes, irrespective of UV-B irradiation, implying that Re and UV-B irradiation, at the conditions used, are nontoxic to HaCaT keratinocytes.

*Attenuation on UV-B-induced proMMP-2 and -9 activities*

**Figure 4:**
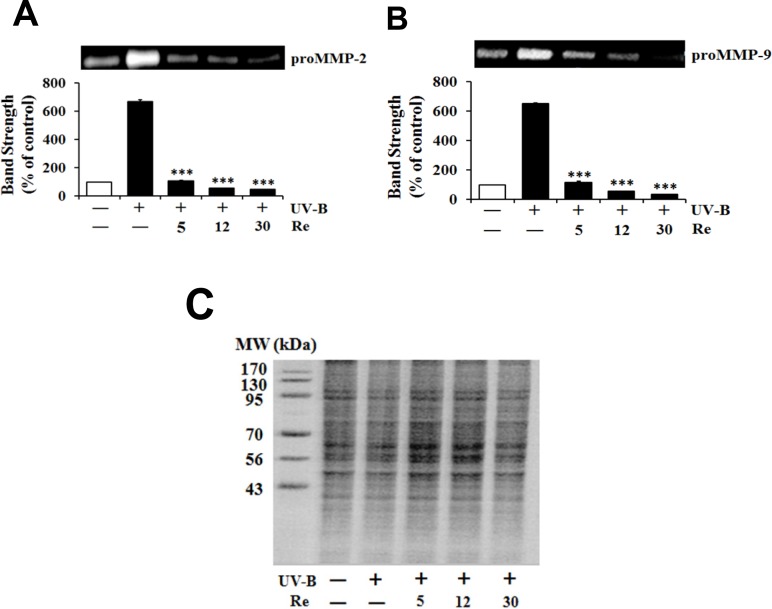
Suppressive effects of Re on pro-matrix metalloproteinase-2 (proMMP-2, A) and -9 (proMMP-9, B) gelatinolytic activity levels in HaCaT keratinocytes under the irradiation with 70 mJ/cm^2^ UV-B. The HaCaT cells were incubated for 24 h in fresh medium, pretreated with the indicated concentrations (0, 5, 12 and 30 μM) of Re for 30 min before the irradiation, and continued to be incubated for further 24 h. The gelatinolytic activities of proMMP-2 and proMMP-9 in conditioned medium, expressed as % of control, were detected using gelatin zymography. In C, the equal loading of conditioned media was shown by the use of Coomassie blue staining of the identical gel. The relative band strength was determined with densitometry using the ImageJ software which can be downloaded from the NIH website. ***P < 0.001 versus the non-treated control (UV-B irradiation only

Since UV radiation, particularly UV-B radiation, is able to enhance production of certain MMP family members, such as MMP-9, -2 and -1, chronic exposure of the skin to UV radiation impairs its normal architecture, leading to skin photoaging and further serious diseases (13). As expected, the UV-B irradiation alone caused the gelatinolytic activities of proMMP-2 and -9 to enhance to 6.7- and 6.5-fold of the non-irradiated values, respectively (Figure 4). When HaCaT cells were pretreated with Re at 5, 12 and 30 μM, the proMMP-2 gelatinolytic activities dropped to 16.3%, 5.9% and 3.9%, respectively, of that from the UV-B irradiation alone (Figure 4A). Re at 5, 12 and 30 μM attenuated the proMMP-9 gelatinolytic activities to 17.9%, 8.6% and 5.6% of that from the UV-B irradiation alone, respectively (Figure 4B). The attenuating effects of Re on both proMMP-2 and -9 activities tend to be proportional depending on its concentrations. These findings, obtained from zymographic analyses, support that Re is capable of attenuating both proMMP-2 and -9 gelatinolytic activities in conditioned medium elevated by UV-B irradiation in HaCaT keratinocytes.

*Diminishment on UV-B-induced proMMP-2 and -9 protein levels*

**Figure 5:**
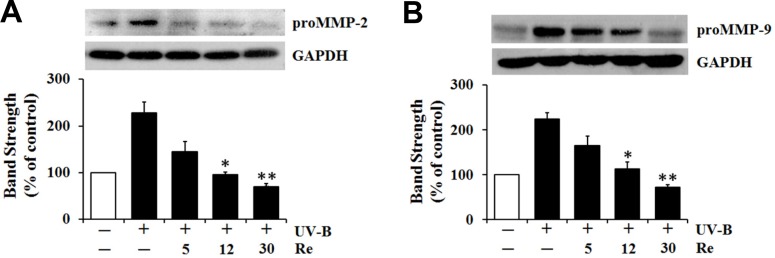
Suppressive effects of Re on pro-matrix metalloproteinase-2 (proMMP-2, A) and -9 (B) protein levels in cellular lysates prepared from HaCaT keratinocytes under irradiation with 70 mJ/cm^2^ UV-B. The HaCaT cells were subjected to the indicated concentrations (0, 5, 12 and 30 μM) of Re for 30 min before the irradiation. The proMMP-2 and -9 proteins, expressed as % of control, were determined using western blotting analysis with anti-MMP-2 and -9 antibodies. GAPDH was used as a protein loading control. The relative band strength, expressed as % of control, was determined with densitometry using the ImageJ software which can be downloaded from the NIH website. **P < 0.01; ***P < 0.001 versus the non-treated control (UV-B irradiation only

Since Re definitely attenuated the UV-B-induced proMMP-2 and -9 gelatinolytic activities in HaCaT cells under UV-B irradiation (Figure 4), the influences of Re on proMMP-2 and -9 protein levels were pursued using western blotting analysis (Figure 5). The UV-B irradiation alone markedly increased proMMP-2 and -9 protein levels in cellular lysates (Figure 5). As shown in Figure 5A, Re, at the concentrations of 5, 12 and 30 μM, could diminish the UV-B-induced proMMP-2 protein levels in cellular lysates to 63.2%, 41.4% and 30.7%, respectively, of that from the UV-B irradiation alone (Figure 5A). Likewise, the UV-B-induced proMMP-9 protein levels in cellular lysates were attenuated by Re in a concentration-dependent manner (Figure 5B). Re at 5, 12 and 30 μM could attenuate the UV-B-induced proMMP-9 elevation to 73.8%, 50.4% and 32.1%, respectively, of that from the UV-B irradiation alone (Figure 5B). Collectively, Re is able to down-regulate the UV-B-induced proMMP-2 and -9 production in HaCaT keratinocytes and subsequently attenuate the UV-B-induced proMMP-2 and -9 gelatinolytic activities in conditioned medium. These results might suggest that Re can weaken the sequential events triggered by UV-B irradiation, such as intracellular ROS elevation, enhanced production and extracellular secretion of proMMP-2 and -9, and increased proMMP-2 and -9 gelatinolytic activities in conditioned medium, which can finally lead to skin photoaging.

*Enhancement on UV-B-reduced total GSH*

**Figure 6.**
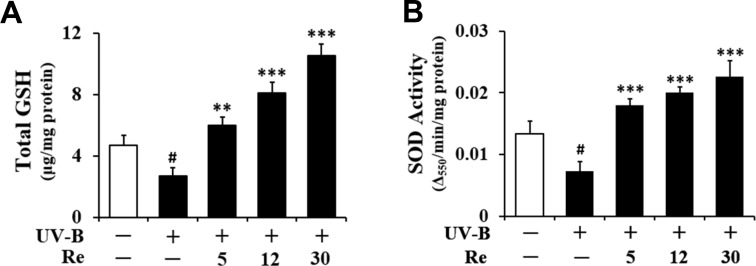
Enhancing effects of Re on total glutathione (GSH, A) and superoxide dismutase (SOD, B) activity levels in cellular lysates prepared from HaCaT keratinocytes under irradiation with 70 mJ/cm^2^ UV-B radiation. The HaCaT cells were subjected to the indicated concentrations (0, 5, 12 and 30 μM) of Re for 30 min before the irradiation. In A, total GSH content, expressed as μg/mg protein, was determined with enzymatic recycling assay using GR. In B, total SOD activity, expressed as Δ_550_/min/mg protein, was measured using a spectrophotometric assay. The relative band strength, expressed as % of control, was determined with densitometry using the ImageJ software which can be downloaded from the NIH website. ^#^*P *< 0.05 versus the non-irradiated control; **P < 0.01, ***P < 0.001 versus the non-treated control (UV-B irradiation only

Consistent with a previous finding (14), total GSH contents were diminished in HaCaT keratinocytes irradiated with 70 mJ/cm^2^ UV-B radiation (Figure 6A). As shown in Figure 6A, the total GSH contents in the irradiated keratinocytes were decreased to 57.6% of the non-irradiated control value. Re at 5, 12, and 30 μM enhanced the UV-B-reduced total GSH contents by 2.2-, 2.9- and 3.8-fold, respectively, compared to that of the irradiated HaCaT keratinocytes that did not receive the treatment (Figure 6A). In brief, these findings indicate that the GSH-enhancing effects of Re might be related to its skin photoaging effects in HaCaT keratinocytes.

*Enhancement on UV-B-reduced SOD activity*

Living cells frequently crash into adverse environments one of which might be an oxidative stressful state. To cope with such challenges, cells are evolutionarily equipped with various kinds of defense systems, including GSH and antioxidant enzymes. SOD, which converts superoxide anion into hydrogen peroxide and molecular oxygen, has been known as one of crucial antioxidant enzymes, since superoxide anion is one of more toxic ROS species. SOD has a key antioxidant role which was also verified by the severe pathologies evident in SOD-knockout mice, including hepatocellular carcinoma, an acceleration of age-related muscle mass loss, an earlier incidence of cataracts, and a reduced life span (15). Total SOD activity in cellular lysates was significantly diminished in HaCaT keratinocytes by the irradiation with 70 mJ/cm^2^ UV-B radiation (Figure 6B). As shown in Figure 6B, the total SOD activities in the irradiated keratinocytes dropped to 54.9% of the non-irradiated control value. This diminishment could be overcome by the pretreatment with Re (Figure 6B). Rb3 at 5, 12 and 30 μM could enhance total SOD activities by 2.5-, 2.7- and 3.1-fold, respectively, compared to that of the UV-B-irradiated HaCaT keratinocytes without the reception of Re treatment (Figure 6B). Taken together, Re is capable of enhancing SOD activity diminished in HaCaT keratinocytes under UV-B irradiation. The SOD-enhancing activity of Re, together with its GSH-enhancing activity, may be an important mechanism for its beneficial effects on the skin.

## Discussion

As people are getting older, they gradually wonder to keep their skin healthy. Since they are exposed to various adverse environmental challenges during normal daily life, such as UV radiation, toxic chemicals, and mechanical wounding, their skin undergoes aging process. Particularly, UV radiation, a component of sunlight, exerts a variety of deleterious effects on human skin, which can lead to diverse types of acute and chronic reactions in skin. The examples of the outcomes include sun burns, photoimmune suppression, photoaging defined as premature skin aging, and skin cancer. Skin photoaging is generally characterized by photooxidative damages with long-term detrimental effects such as wrinkles, loss of skin tone and resilience. Hyperpigmented lesions, also known as age spots, are one of the most visible alterations in skin photoaging. Until recently, many researchers have devoted to seek effective strategies to prevent and attenuate skin photoaging. 

Among the components of solar UV radiation, the UV-B (290-320 nm) component is highly mutagenic and carcinogenic, which was previously identified using animal models (16), compared to the UV-A (320-400 nm) component. Toxic effects of UV radiation, a potent inducer for skin photoaging, on human skin include protein/lipid oxidation and DNA damage at molecular levels, the latter of which plays a major role in photocarcinogenesis and photoaging. During skin photoaging process, proteins are damaged by ROS, resulting in accumulation of oxidatively modified protein. UV irradiation generates irreversible oxidation of the side chains of certain amino acids, resulting in the formation of carbonyl groups on proteins. However, certain amino acid oxidation products such as methionine sulfoxide can be reversed back to the reduced forms within proteins by specific repair enzymes, such as methionine sulfoxide reductases A and B (17). The unifying pathological agents for skin photoaging are currently believed to be UV-generated ROS which can deplete and damage naturally equipped non-enzymatic and enzymatic antioxidant defense systems of the skin, leading to adverse changes (18). Since skin photoaging results from the co-working of both cutaneous oxidative stress and inflammation caused by UV radiation, UV radiation is widely considered as a major cause of human skin photoaging and even skin cancer. UV radiation-induced pro-inflammatory cytokines mediated by NF-κB reportedly play important roles in photoaging and cancer (19). In this work, we used two fluorescent ROS probes, DCFH-DA and DHE, which are known to react with different ROS species, to detect the changes in intracellular ROS levels. Both probes proved significantly enhanced ROS levels in the UV-B-irradiated HaCaT keratinocytes. This might correspond with the previous finding that UV-B radiation immediately produces superoxide anion which is sequentially converted to other species of ROS in epidermal keratinocytes (20). Re was capable of scavenging the UV-B-induced ROS in a concentration-dependent manner, which was also proved by two different fluorescent probes. This antioxidative activity of Re could be related with blocking the development of oxidative stress conditions in keratinocytes under UV-B irradiation.

MMPs, a complex family of zinc-containing proteinases, are capable of degrading essentially all components of extracellular matrix forming skin dermal connective tissue (21). Since MMP-2 and -9 gelatinolytic activities can degrade types IV and VII collagens, the principal components of the epidermal basement membrane, UV-induced gelatinolytic activities play a prominent role in the UV-induced skin damaging process (22). Some MMPs are produced and released by cells in the precursor forms (proMMPs), and their activation is controlled by a cascade of steps involving other MMPs and the plasmin system (23). Excessive ROS under oxidative stress in conjunction with the resulting inflammation up-regulate the over-expression of MMPs, which in turn causes the degradation of extracellular matrix, finally leading to coarse wrinkling, dryness, and laxity of the skin (24). UV-B radiation was previously found to significantly enhance proMMP-2 and -9 mRNAs and gelatinolytic activities in HaCaT keratinocytes (25). 

Since MMPs directly participate in the degradation of extracellular matrix proteins, their levels are very crucial in skin photoaging. ROS is considered as a major factor to initiate the up-regulation of MMPs in keratinocytes and fibroblasts via the activation of receptor proteins on the cell membrane of those cells, and to degrade fiber components in dermis, leading to wrinkle formation (26). UV-B-induced ROS activate latent transforming growth factor-β complex by stimulating MMP-2 and -9 activities (27). (2’S)-Columbianetin, isolated from a salt tolerant plant and known to have antioxidant and anti-inflammatory activities, suppresses ROS generation, cell cycle arrest at sub-G1 and induction of MMP-2 and -9 in the UV-B-irradiated HaCaT keratinocytes (28). T-2 toxin, one of the most toxic among several trichothecenes involved in both human and animal poisoning, induces skin inflammation and cutaneous injury which are mediated through ROS generation and subsequent activation of MMP-2 and -9 (29). Enzyme-treated ginseng leaf extract suppresses ROS generation, GSH depletion, and expression of MMP-2 and -9 induced by UV-A irradiation in HaCaT keratinocytes (30). As described above, several components or mixtures were shown to simultaneously down-regulate both MMP-2 and -9 under certain stress conditions. Likewise, in this work, the UV-B-induced proMMP-2 and -9 appeared to be down-regulated by Re at production and secretion levels possibly via a common mechanism. Although proMMP-2 and -9 have been shown to be co-regulated at least in some cell types, their common mechanism and its precise physiological meaning remain elusive.

With examining the abilities to scavenge several reactive species associated to UV-induced oxidative stress, many plants extracts and natural compounds have been recognized as photoprotective agents that can diminish the UV-induced ROS elevation. There have also been several examples which display their scavenging effects in the skin cells through modulating antioxidant components. Diphlorethohydroxycarmalol, a phlorotannin isolated from an edible brown algae and known to exhibit antioxidant effect and exert protective actions against UV-B radiation and γ ray radiation (31,32), was found to restore cellular viability reduced by UV-B radiation, decrease UV-B-induced ROS, increase levels of reduced GSH, activate SOD and catalase in HaCaT keratinocytes (33). These findings imply that diphlorethohydroxycarmalol safeguards human keratinocytes against UV-B-induced cell damage by absorbing UV-B radiation, scavenging ROS and enhancing antioxidant components (33). An ethanol extract of hawk tea, used as a folk medicine to prevent and treat gastrosis, hepatitis and some other inflammatory diseases, exhibits a protective activity against UV-B-induced oxidative stress in HaCaT keratinocytes through the inhibition of lipid peroxidation, reduction of ROS levels and stimulation of antioxidant enzymes activities, such as catalase, SOD and GSH peroxidase (34). Glyphosate, known as a herbicide, induces significant changes in cellular antioxidant status as a GSH depletion, enzymatic disorders, including catalase, GSH peroxidase and SOD, and increased lipid peroxidation, which can be protected by vitamin C and vitamin E (35).

This work demonstrates that Re is capable of enhancing the UV-B-reduced total GSH contents and SOD activities. This finding might imply that Re possesses indirect antioxidative properties via up-regulating antioxidant components, including GSH and SOD, which might be a preceding step in the antioxidative action of Re. However, we can also think an unlikely possibility that antioxidant components are accumulated based on a sparing effect due to a presumed potent antioxidative properties of Re. None of the current findings supports this less favorable possibility.

In conclusion, the potential skin anti-photoaging activity of Re was clarified in HaCaT keratinocytes subjected to UV-B radiation. Re suppresses the UV-B-induced intracellular ROS generation and production and secretion of proMMP-2 and -9, while it enhances the UV-B-reduced total GSH levels and SOD activity. Re may elicit its skin anti-photoaging property through down-regulation of the UV-B-induced proMMP-2 and -9 in an ROS-dependent mechanism, which results from the overcoming of total GSH and SOD activity diminished under UV-B irradiation. Ginsenoside Re can be a plausible candidate utilized as a natural resource for manufacturing improved anti-photoaging cosmetics with fewer side effects.
